# Unexpected giant cell aortitis in a young patient

**DOI:** 10.1016/j.ijscr.2021.105741

**Published:** 2021-03-11

**Authors:** Lawrence Nair, Eshan Senanayake, Bruce Thomson

**Affiliations:** Department of Cardiothoracic Surgery, The Prince Charles Hospital, Brisbane, Queensland, Australia

**Keywords:** Giant cell, Aortitis, Aortopathy, Cardiac surgery, Aneurysm

## Abstract

**Introduction and importance:**

Giant cell aortitis is a rare cause of ascending aortic aneurysm disease. Patients presenting with aneurysms caused by giant cell aortitis are at high risk of devastating complications.

**Case presentation:**

A 35-year-old female with an ascending aortic aneurysm, underwent an aortic root and ascending aorta replacement and subsequently was found to have giant cell aortitis on histopathology.

**Discussion and conclusion:**

This case illustrates a very rare cause for aortopathy in a young healthy patient, who may have ruptured or dissected, if not for timely operative intervention.

## Highlights

• Giant cell aortitis is a rare cause of aortic aneurysm and the incidence of this in a young patient with no prior cardiovascular risk factors remains unknown.
• Patients presenting with aneurysms caused by giant cell aortitis are at high risk of devastating complications.
• Intraoperative tissue assessment played a valuable role in providing a secure long-term durable outcome.

## 1 Introduction

Giant cell aortitis is a rare cause of ascending aortic aneurysm disease. The incidence of this in a young patient with no prior cardiovascular risk factors remains unknown. Patients presenting with aneurysms caused by giant cell aortitis are at high risk of devastating complications. Liu and colleagues [1] described a series of 23 patients with giant cell aortitis presenting with aortic dissection, 46 % of patients presenting in extremis and a 2-week mortality within this group of 80 %. Histologic features suggest that there is often near-complete disruption of the elastic medial layer [2] as demonstrated in this case. This disruption could predispose patients to rupture and dissection before an aneurysm achieves the standard size criteria for intervention. This case report is in line with SCARE 2020 criteria [3].

## 2 Clinical record

A 35-year-old female with an ascending aortic aneurysm, underwent an aortic root and ascending aorta replacement and subsequently was found to have giant cell aortitis on histopathology. She had been monitored for a known cardiac murmur since childhood however had no clinical symptoms nor significant family history of cardiovascular disease. Transthoracic echocardiogram demonstrated mild aortic incompetence and the presence of a dilated ascending aorta, measuring 57 mm in diameter. Computed tomography demonstrated an ascending aortic aneurysm measuring 51 mm x 58 mm with a normal sinotubular-junction and an aortic root with normal dimensions and morphology.

**Fig. 1:**
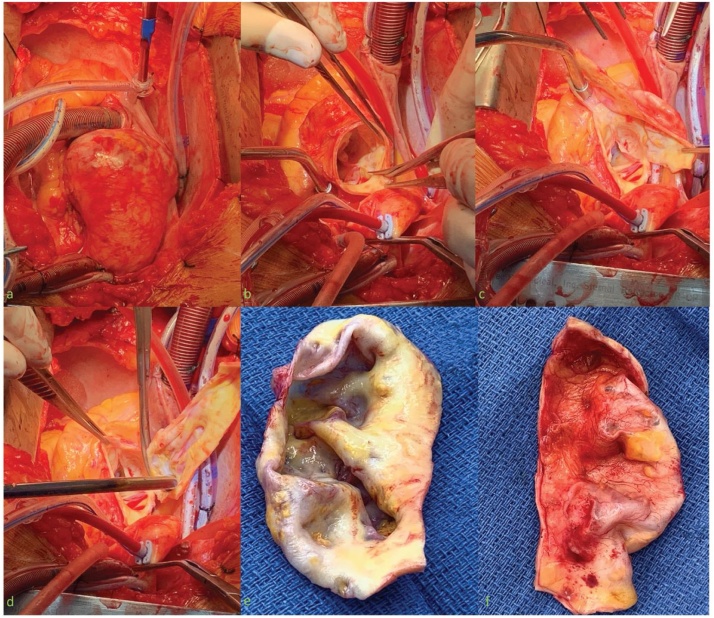
a–d) Intraoperative findings of aortic root and ascending aorta, e,f) ulcerated wall of ascending aorta.

**Fig. 2:**
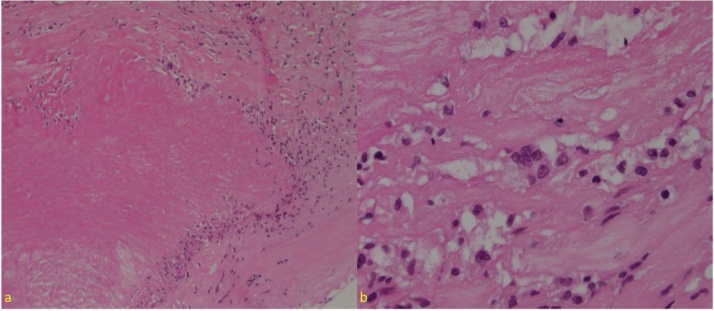
a) Myxoid medial degeneration with multinucleated giant cell, b) multinucleated giant cell (in centre).

The patient underwent a mechanical aortic root and ascending aorta replacement by the attending surgeon. Intraoperatively the ascending aorta was noted to be dangerously large, with features suggesting pre-rupture (Fig. 1a). The aortic wall was thin and ulcerated (Fig. 1e,f), with thinning of the aortic root and sinuses. As a consequence, due to the poor integrity of the aortic root tissue and uncertainty of the aetiology causing extensive ulceration of the ascending aorta in a young patient, an aortic root and ascending aorta replacement was performed with a 25 mm mechanical composite valved conduit. Post-operative recovery was unremarkable, and the patient discharged home seven days following the procedure. Histopathology of the diseased aorta demonstrated an aortitis characterised by patchy laminar necrosis of the aortic media with surrounding lymphocytes, palisaded histiocytes and scattered multinucleated giant cells (Fig. 2). Some of the necrotic foci were infiltrated by foamy histiocytes and were associated with a transmural inflammatory infiltrate, including plasma cells and eosinophils in addition to the lymphocytes. In areas where the aortic wall was at its thinnest, there was complete destruction of the aortic media, consequently replaced by dense hyalinised fibrosis. There were no features to suggest infective aortitis.

## 3 Conclusion

In a young patient with no risk factors for aortopathy one has to be suspicious of the aetiology of aneurysmal dilatation. In this case, despite normal root dimensions and morphology, the poor integrity of the root tissue compounded by the unknown aetiology at the time, necessitated a root replacement for long-term prognostic benefit. This case illustrates a very rare cause for aortopathy in a young healthy patient, who may have ruptured or dissected, if not for timely operative intervention.

## Conflicts of interest

Nil.

## Sources of funding

Nil funding.

## Ethical approval

N/A.

## Consent

Written informed consent was obtained from the patient for publication of this case report and accompanying images. A copy of the written consent is available for review by the Editor-in-Chief of this journal on request

No identifying characteristics/features are noted throughout the case report

## Author contribution

Equal contribution by all authors

## Registration of research studies

N/A.

1 Name of the registry:
2 Unique identifying number or registration ID:
3 Hyperlink to your specific registration (must be publicly accessible and will be checked):

## Guarantor

The first author accepts full responsibility for the work provided

## Provenance and peer review

Provenance and peer review Not commissioned, externally peer-reviewed.
